# Unraveling the Significance of Draglines: Female Sexual Signalization in the Nursery-Web Spider, *Pisaura mirabilis*

**DOI:** 10.3390/insects14090765

**Published:** 2023-09-13

**Authors:** Zuzana Ježová, Pavol Prokop, Martina Zvaríková, Milan Zvarík

**Affiliations:** 1 Department of Environmental Ecology and Landscape Management, Faculty of Natural Sciences, Comenius University in Bratislava, Ilkovičova 6, 842 15 Bratislava, Slovakia; zuzana.jezova@uniba.sk (Z.J.);; 2 Institute of Zoology, Slovak Academy of Sciences, Dúbravská Cesta 9, 845 06 Bratislava, Slovakia; 3 Department of Nuclear Physics and Biophysics, Faculty of Mathematics, Physics and Informatics, Comenius University, Mlynská Dolina F1, 842 48 Bratislava, Slovakia; milan.zvarik@uniba.sk

**Keywords:** chemical signalization, *Pisaura mirabilis*, draglines

## Abstract

**Simple Summary:**

Various signals are used in insects to identify mates. Signals based on visual, acoustic, or chemical communication play an important role in sexual selection. Frequently occurring chemical-based signals are the most important and understudied field of communication. Spiders use a wide variety of chemicals to find potential mates, but, until now, only a few spider-used pheromones have been identified. The nursery-web spider, *Pisaura mirabilis*, does not build a typical net, but leaves draglines on the ground. In our research, we investigated the role of chemical signals in female draglines influenced by factors such as spider ontogeny, nutritional status, and female mating status. Our findings indicate that the chemical signals of eggsac-carrying females were similarly sexually attractive to females not carrying eggsac. Draglines of adult and hungry females functioned, in contrast, as stimulation for male motivation to invest more silk in gift production. Chemical differences in female draglines were identified, but they did not always correspond with male behavior. We suggest the integration of behavioral and chemical approaches to better understand animal behavior in future research.

**Abstract:**

Chemical signals used by animals to attract the opposite sex are well known in insects, but heavily understudied in spiders. We investigated the role of chemical signals in female draglines in a gift-giving spider, *Pisaura mirabilis*, using combined data from behavioral tests and high-performance liquid chromatography (HPLC). We also investigated whether the quality of sexual signalization is influenced by crucial factors, such as female spider ontogeny, nutritional status, and mating status. We found that draglines of adult (versus subadult) and hungry (versus fed) females stimulated male motivation to produce nuptial gift, and highly sexually excited males invested more silk in gift production than less sexually excited males. Unexpectedly, chemical signals of eggsac-carrying females were similarly sexually attractive to draglines of adult females not carrying eggsac. HPLC identified significant chemical differences in female draglines, but these differences did not always correspond to male behavior. The integration of behavioral and chemical approaches is required to better understand animal behavior in future research.

## 1. Introduction

Invertebrates use a variety of cues to identify potential mates, including visual, auditory, and chemical signals. These signals play an important role in sexual selection in many invertebrate species [1,2,3,4,5]. Males can produce complex and species-specific signals to attract females and compete with other males for mating opportunities. Females, on the other hand, attract males and select the most appropriate male(s) as mate(s) [6]. Chemical signals, or pheromones, are particularly important in sexual selection in invertebrates for their often-occurring species-specific ability of recognition and detection at low concentrations over long distances [7].

Since Darwin’s time, scientists have conducted extensive research on the role of pheromones in the choice of mate in invertebrates. They have identified specific pheromones and pheromone receptors in various invertebrate species and have shown that these signals play a critical role in guiding mate choice and reproductive behavior [8]. However, traditional research of sexual selection has mainly been based on visual signals or acoustic signals in communication [9,10].

The complex chemical signaling that occurs during spider courtship is essential for their reproductive activity. Chemical signaling enables spiders to differentiate between species and, by detecting and responding to chemical cues, avoid energetically costly interactions with individuals from different species [11]. The complexity and specificity of the chemical cues used in communication is unique in each species [12] and may indicate condition, age, fertility, mating status, or the body size of individuals [13,14,15,16,17,18,19]. Although Andersson [6] ranked chemical signaling as the third most important animal communication route, we still know very little about chemical communication among spiders [20], and only 11 spider pheromones have been identified so far [21,22]. Pheromone production helps females engage in energy-efficient strategic advertising and reduces the overall energy costs of mating interactions [23]. Finding a compatible partner is the first stage in successful reproduction, and due to solitary living, it may not be straightforward [24,25]. Mechanical signals such as following silk lines left on the substrate, as was observed in the crab spider *Misumena vatia* (Clerck, 1757), are errors liable in mating for following very old drag lines as well as new ones [26]. Some pheromones (released from the web, cuticular) are emitted as airborne volatiles, while others are detected only by direct contact [27]. A series of experiments were made on *Portia labiata* (Thorell, 1890) that suggested that spiders of this species use a more advanced system of communication through pheromones. Females can recognize between their own eggsacs, draglines, and draglines from those of members of the same species. They can also differentiate between draglines of unknown or antagonistic females [28,29]. Once a male approaches a conspecific female, there is a wide variety of opportunities for successful courtship signals to control predatory females’ hunting behavior.

This requires males to identify themselves as the same species while attracting the female’s attention [23].

*Pisaura mirabilis* presents an interesting system to study chemical signals through the spiders’ silk. Nuptial gifts given by males to females during courtship and the consumption of gifts wrapped in silk during copulation are features found in webless gift-giving spiders. Silk is utilized for more than just gift-wrapping; both sexes also use it as draglines when moving, to locate mates and improve the probability of finding them. When the dragline of a female *P. mirabilis* appears on the substrate, the male responds by shaking his opisthosoma and following the dragline until he reaches the female [30,31,32,33]. Pheromones related to female silk are considered to induce sexual arousal in males, which is also exhibited as leg-rubbing and gift-wrapping behavior [30,31,34]. According to numerous studies, female draglines include chemical information that stimulates male spiders to begin wrapping a gift in silk to become sexually excited [31,35,36,37,38]. The latest study from Eberhard et al. [30] found reduced courtship in response to the subadult female dragline in performing the vibrational part of courtship after contact with silk. Silk wrapping of nuptial gifts may generally provide information about males to females through visual, tactile, or chemical cues [39]. In theory, the brightness and chemical composition of the wrapped silk can provide information about the male condition to the female, because starved males use less silk in gift production than well-fed males [37]. In certain species, for instance, pheromones can provide information about the body condition of a male [37,40,41], just like the amount of silk used for the nuptial gift [37,42]. Some indirect evidence suggests that silk cover can not only prolong copulation [31,36], but also may contain chemical compounds that can potentially manipulate female reproductive behavior [34]. By depositing additional layers of silk, males may be adding chemical cues to persuade reluctant females to mate above their optimal mating rates or by providing information about their inherent qualities to aid females in selecting a mate [34].

Diet influences the condition of the individuals and, consequently, the potential to synthesize pheromones [43]. Well-fed individuals are generally more attractive than starved individuals [44,45,46]. In species with nuptial feeding, however, such as *P. mirabilis*, male donations may enhance female fecundity [47,48,49]; thus, food-limited females engage in more matings than satiated females [48,49,50,51,52,53,54,55]. We hypothesize that starved females of nuptial feeding species, receiving nutritious gifts from males, increase their pheromone output, leading to a more sexually attractive signal.

In this paper, we combined behavioral research with data obtained with high-performance liquid chromatography (HPLC) to investigate the role of draglines in sexual signaling in *P. mirabilis*. The inability to reproduce by juvenile females and the high mating rates of hungry females suggest that males can discriminate between juvenile and adult females (H1), as well as between hungry and well-fed females (H2), based on chemical signals in female draglines. Consistent with these hypotheses, HPLC analyses are expected to show differences between draglines of juvenile and adult females (H3) and between draglines of hungry and well-fed females (H4). Females carrying eggsacs do not mate, and males die in this time of the mating season [56]. We therefore hypothesize that draglines produced by eggsac-carrying females are not sexually attractive for males (H5) and there are chemical differences in draglines between adult, sexually receptive, and adult, but eggsac-carrying and, thus, sexually unreceptive, females (H6). 

## 2. Materials and Methods

### 2.1. Study Organism

*Pisaura mirabilis*, commonly known as the nursery-web spider and belonging to the Pisauridae family, is a frequently encountered spider found in deciduous forests, meadows, and abandoned grasslands. In Central Europe, spiderlings hatch during the summer and reach maturity in the subsequent spring [57]. Mating typically occurs from mid-April to late May, with timing dependent on climatic conditions (P. Prokop, personal observations). Following the end of the mating season, males die, while females engage in maternal care for both eggs and spiderlings [58].

Sexually receptive females release draglines containing pheromones that attract males. Upon detecting these draglines, males become sexually aroused, capture a prey item, and encase it in silk [31,32,37,38,59]. Subsequently, the male approaches the female, and she grasps the gift with her chelicerae. During this interaction, the male inserts either his right or left pedipalp into the female’s epigyne, transferring sperm while the female consumes the gift [60].

### 2.2. Collection and Maintenance

In April–May 2021, a total of 100 male and 100 female subadult spiders were collected from various grasslands and small woods near Trnava, Slovakia (N 48°37, E 17°58). Each spider was individually housed in a ventilated 0.3 L glass jar containing wet cotton to maintain humidity. The jars were placed outdoors on private property in Trnava, Slovakia (48°23′ N, 17°35′ E), sheltered from direct sunlight and subjected to the natural photoperiod and temperature. The spiders were sprayed with water daily and provided with ad libitum feeding three times per week with dead house crickets (*Gryllus assimilis*), approximately one adult cricket per feeding. Of the initial sample, 95 males (with an additional five males that died) and 94 females (with an additional six females that died) molted into adulthood during May 2021. After the experiments ended, all the spiders were released near their original capture sites by early June 2021.

### 2.3. Female Pheromones/Gift Production Experiment

Approximately 10–15 days after molting, each male spider was randomly assigned to one of the following treatments: subadult female pheromones (N = 15), hungry adult females (N = 33, 14 of them were nonvirgin), and fed adult females (N = 36, 1 of them was nonvirgin). Most of the adult females used in the experiment were virgin (N = 54), while some were intentionally mated (hereafter nonvirgin), with males not included in this particular study (N = 15). In addition, a final treatment group consisted of females carrying eggsacs (N = 9). On the day of the experiment, all the individuals were anesthetized with CO_2_, weighed on an analytical balance to the nearest 0.0001 g, and the width of the prosoma was measured with digital calipers (to 0.01 mm).

The hungry and fed adult females received ad libitum food during the first five days after reaching adulthood. Fed females continued to receive ad libitum food for the next ten days, while hungry females were not given any food and remained in a starved state following this [55].

To manipulate the presence of female pheromones, a female spider was randomly placed in a glass terrarium (30 × 20 × 20 cm) lined with clean white paper for 20 min. The mobility of both sexes was evaluated according to Kotiaho et al. [61] and Prokop and Semelbauer [62]. The females were assessed in the absence of the males. Briefly, the terrarium was divided into quarters, and the number of times a male entered a new section of the arena was recorded as the mobility index. The mobility of the male was measured using the same method after the female was removed from the terrarium, i.e., in the presence of female pheromone cues. Mobility was measured only once due to the moderate repeatability of male mobility in *P. mirabilis* in three trials (R = 0.30) [59]. Higher mobility index values indicated greater mobility. Measurements of mobility were used as an estimate of the quantity of draglines produced and subsequently controlled in statistical analyses. There were no significant effects of the level of hunger of the female (fed or hungry), mating status (virgin or nonvirgin), developmental stage (adult or subadult), or the presence of maternal care (having an eggsac or not) on female mobility (one-way ANOVAs, all *p* > 0.28).

After the female released the draglines, the male was placed inside and allowed to acclimate for two minutes. Visual communication between males and females was not allowed. A dead cricket nymph weighing approximately 0.02 g (which falls well within the range of gifts found in this locality, cf. Prokop and Maxwell [63], Prokop and Semelbauer [62]) was then positioned about 1 cm in front of the male’s chelicerae. The male’s behavior towards the dead cricket nymph was observed for a duration of ten minutes, including prey carrying or ignoring and male mobility. Male investment in nuptial gift production was measured as the total time males spent wrapping a gift in silk (in seconds), because this variable significantly correlates with the total amount of silk produced [64]. Additionally, we documented specific behaviors that indicate male sexual excitement, including trembling of the palps and abdomen, jerking of the body, and rapid rubbing of the legs [31,36]. The presence of any of these three behaviors during the trial was coded as 1, while their absence was coded as 0. The sum of these scores (minimum = 0, maximum = 3) was utilized as the sexual excitement index in the statistical analyses. Higher scores indicated a greater intensity of sexual excitement. The sample sizes for the chemical analyses were the same as for the behavioral tests.

After the trial, each female was placed in a new terrarium. Using sterile gloves, we forced each female to jump from a piece of filter paper held in hands in order to stimulate dragline production. The dragline was immediately wrapped around the filter paper 30 times. The width of the filter paper was 50 mm. The samples were stored in an Eppendorf tube filled with 1 mL distilled water.

### 2.4. Sample Preparation

Each sample (dragline in distilled water) was mixed at the room temperature (21 ± 1 °C) for 24 h. Subsequently, the extract was collected, filtered through a 200 nm filter, and subjected to HPLC analysis.

### 2.5. HPLC Analysis

The chromatographic analysis was performed in gradient mode, using water and acetonitrile (ACN) as mobile phases at a flow rate of 0.5 mL/min. Initially, the mobile phase consisted of a 20% aqueous solution of ACN. From the 30th second onwards, the proportion of ACN increased linearly until it reached 100% at the 150th second. From that point until the end of the measurement, the mobile phase consisted of 100% acetonitrile. An Agilent Poroshell 300 SB-C18 column (1 × 75 mm, 5 um) was used for the separation. The injected sample volume was 10 µL, and the measurements were carried out at a temperature of 25 °C. An absorption detector and a fluorescence detector were used for signal detection. The absorption detector detected signals at four selected wavelengths—215, 250, 280, and 340 nm. The fluorescence signal (emission at 360 nm) was detected at two excitations—250 and 280 nm.

### 2.6. Statistical Analyses

Behavioral data were analyzed with a generalized linear model (GLM). The GLM with binomial distribution was used to examine the production of nuptial gift (yes = 1, no = 0), and Tweedie distribution was used to examine male investment in gift production. Statistical analyses of HPLC data were performed using R software. For intergroup comparison, the areas under each chromatographic peak were compared using the *t*-test or the nonparametric alternative Mann–Whitney U-test. In the case of evaluating the influence of the sexual excitement index (0–3) on the areas under the peaks, we used analysis of variance (ANOVA) and ordinal logistic regression.

## 3. Results

### 3.1. Biometry of Fed and Hungry Females

The body condition of the fed females on the day of the mating trial differed significantly from that of hungry females. Body condition was assessed through ANOVA [65], with body mass as a dependent variable, treatment as a categorical factor (F1,63 = 86.19, *p* < 0.001) and prosoma width as a covariate (F1,63 = 57.75, *p* < 0.001).

Pairwise comparisons revealed that the fed females (mean = 0.189 g, SE = 0.005, N = 33; three additional females were removed due to missing data) were significantly heavier than females from the hungry treatment (mean = 0.127 g, SE = 0.005, N = 33) (Tukey post hoc test, *p* = 0.0001). There were no differences, in contrast, in male body mass between treatments (fed treatment, mean = 0.089 g, SE = 0.003, N = 33; hungry treatment, mean = 0.099 g, SE = 0.002, N = 33) (F1,63 = 1.88, *p* = 0.17). Only the width of the prosoma significantly influenced the body mass of the male (F1,63 = 170.23, *p* < 0.0001). These results show that female body condition was significantly influenced by starvation.

### 3.2. Behavioral Evidence to Trigger Male Sexual Behavior by Chemical Signals

There were significant differences in the probabilities of accepting dead prey by males between treatments (Fisher exact test, *p* < 0.001). The prey was accepted most frequently, albeit unexpectedly, in eggsac treatment (100%, 9/9), followed by treatment of sexually receptive adult females (53%, 35/66), and less frequently in sexually unreceptive subadult females (13%, 2/15). Nuptial gifts were produced in 44% (4/9) of the eggsac treatment, followed by treatment of sexually receptive adult females (18%, 12/66) and in sexually unreceptive subadult females (7%, 1/15). Differences in nuptial gift production did not differ significantly, however, between treatments (Fisher exact test, *p* = 0.073). Considering specifically sexually receptive adult females (i.e., females with eggsacs and subadult females excluded), logistic regression showed that sexually excited males produced gifts more frequently than less excited males. The draglines of hungry females (27%, 9/33) also stimulated males to produce gifts more than the draglines of well-fed females (9%, 3/33) (Table 1). Draglines of virgin females (18%, 9/51) stimulated males to produce nuptial gifts at similar rates to those of draglines of mated females (20%, 3/15). The mobility of the male and female did not influence the probability of the nuptial gift production (Table 1).

### 3.3. Male Investment in the Production of Nuptial Gifts

More sexually excited males invested more time in gift production than less sexually excited males (Table 2). Other variables were not significant.

### 3.4. HPLC Analyses of Female Draglines

**Figure 1:**
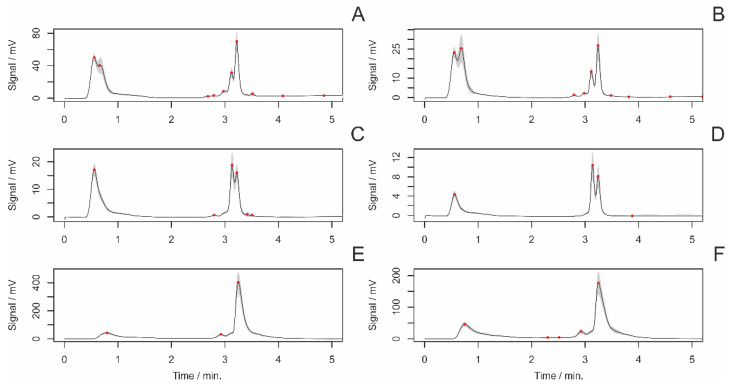
The average chromatograms of chemical substances from the surface of fibers at various settings of the absorbance detector (AD) and fluorescent (FD) detector. The settings of the detectors were as follows: (**A**)—AD 215 nm, (**B**)—AD 250, (**C**)—AD 280 nm, (**D**)—AD 340 m, (**E**)—FD 360 nm (ex. 250 nm), (**F**)—FD 360 nm (ex. 280 nm). Red dots indicate evaluated peaks. The grey area represents a 95% confidence interval.

The average chromatograms of chemical substances extracted from the fiber surface are shown in Figure 1. The intensity of individual peaks was determined from the respective chromatograms, representing specific chemical substances or groups of substances. In total, 43 peaks were identified and subjected to statistical analysis.

#### 3.4.1. Differences between Adult and Subadult Individuals

**Figure 2:**
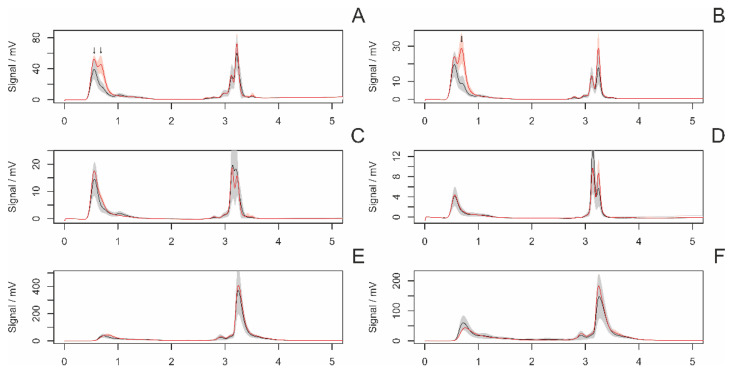
The average chromatograms of extracted chemical substances from the surface of fibers in adult (red) and subadult (black) females, along with their 95% confidence intervals. The settings of the detectors were as follows: (**A**)—AD 215 nm, (**B**)—AD 250, (**C**)—AD 280 nm, (**D**)—AD 340 m, (**E**)—FD 360 nm (ex. 250 nm), (**F**)—FD 360 nm (ex. 280 nm). The gray area represents a 95% confidence interval. Arrows indicate significant peaks in the intergroup comparison.

**Figure 3:**
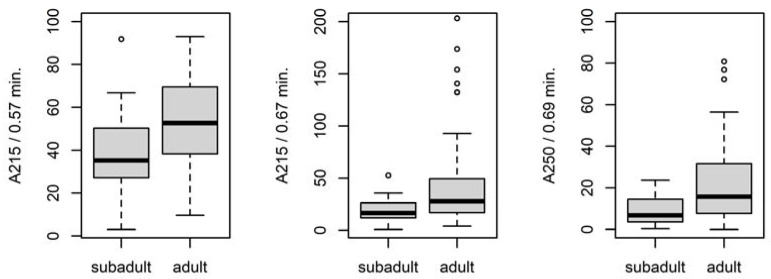
A comparison (boxplots) of statistically significant (*p* < 0.05) peaks in samples of draglines from adult and subadult females. The *y*-axis represents the signal of individual peaks. Labeling of peaks: method/retention time.

Figure 2 displays the average chromatograms of fiber samples from adult and subadult females. The statistical analysis revealed a significant difference in three peaks (Figure 3).

#### 3.4.2. Differences between Virgin and Nonvirgin Females

**Figure 4:**
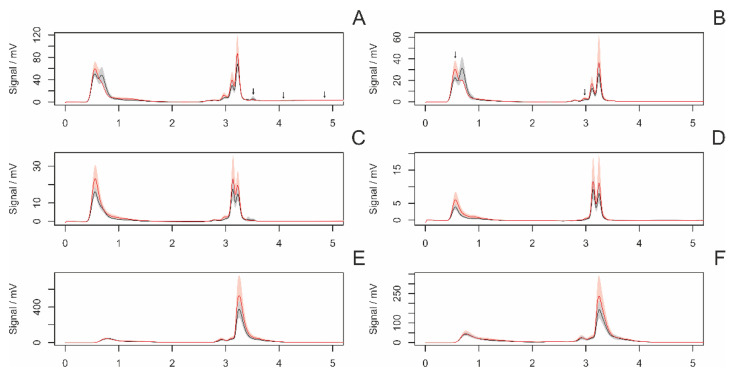
Average chromatograms of extracted chemical substances from the fiber surface in nonvirgin (red) and virgin (black) females, along with their 95% confidence intervals. The settings of the detectors were as follows: (**A**)—AD 215 nm, (**B**)—AD 250, (**C**)—AD 280 nm, (**D**)—AD 340 m, (**E**)—FD 360 nm (ex. 250 nm), (**F**)—FD 360 nm (ex. 280 nm). Arrows indicate significant peaks in the intergroup comparison.

**Figure 5:**
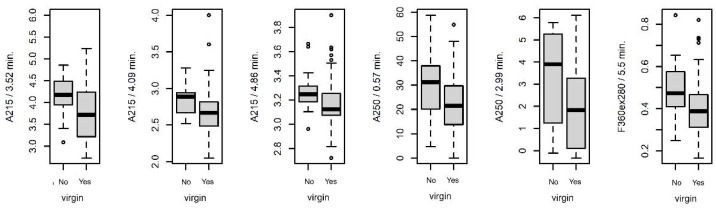
A comparison (boxplots) of statistically significant (*p* < 0.05) peaks in samples of draglines from virgin and nonvirgin females. The *y*-axis represents the signal of individual peaks. Labeling of peaks: method/retention time.

Figure 4 presents the average chromatograms of fiber samples from virgin and nonvirgin females. The statistical analysis revealed a significant difference in six peaks (Figure 5).

#### 3.4.3. Females with and without Eggsacs

**Figure 6:**
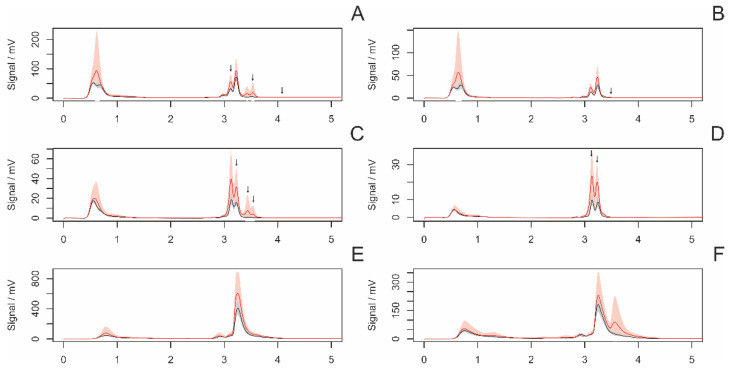
The average chromatograms of extracted chemical substances from the fiber surface in females with an eggsac (red) and without an eggsac (black), along with their 95% confidence intervals. The settings of the detectors were as follows: (**A**)—AD 215 nm, (**B**)—AD 250, (**C**)—AD 280 nm, (**D**)—AD 340 m, (**E**)—FD 360 nm (ex. 250 nm), (**F**)—FD 360 nm (ex. 280 nm). Arrows indicate significant peaks in the intergroup comparison.

**Figure 7:**
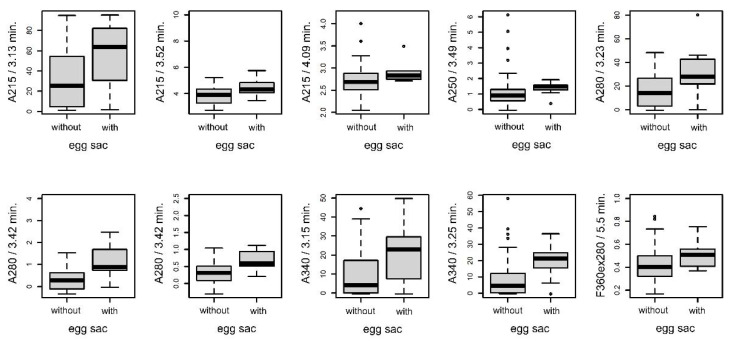
Comparison (boxplots) of statistically significant (*p* < 0.05) peaks (*p* < 0.05) in dragline samples from females with and without eggsacs. The *y*-axis represents the signal of individual peaks. Labeling of peaks: method/retention time.

Figure 6 shows the average chromatograms of fiber samples from females with and without an eggsac. The statistical analysis revealed a significant difference in up to ten peaks (Figure 7).

#### 3.4.4. Hungry and Fed Females

**Figure 8:**
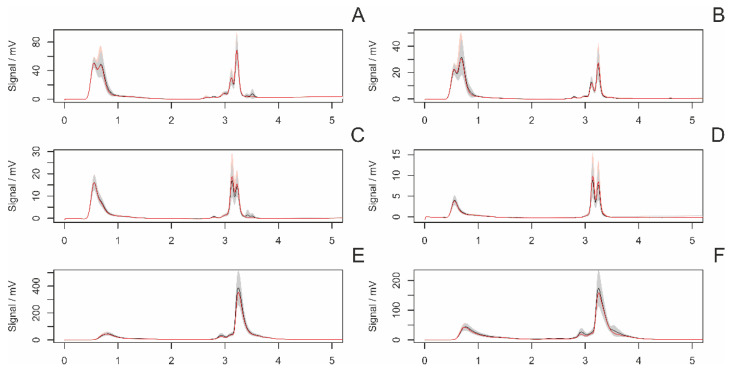
The average chromatograms of extracted chemical substances from the surface of fibers in hungry (red) and fed (black) females, along with their 95% confidence intervals. The settings of the detectors were as follows: (**A**)—AD 215 nm, (**B**)—AD 250, (**C**)—AD 280 nm, (**D**)—AD 340 m, (**E**)—FD 360 nm (ex. 250 nm), (**F**)—FD 360 nm (ex. 280 nm).

Figure 8 presents the average chromatograms of fiber samples from hungry and fed females. The statistical analysis did not reveal a significant difference in any of the observed peaks.

#### 3.4.5. Sexual Excitement Index

**Figure 9:**
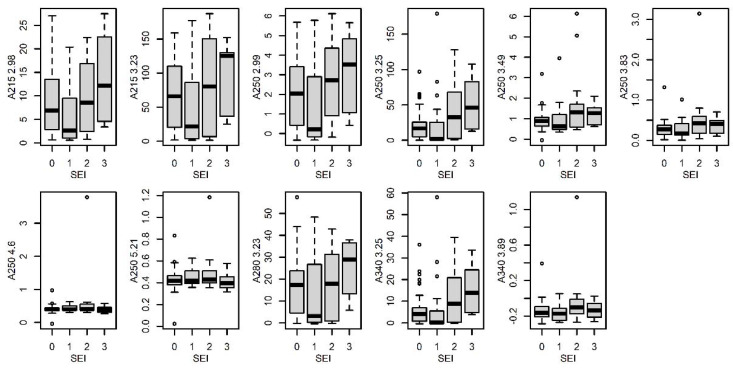
Comparison (boxplots) of statistically significant (*p* < 0.05) peaks in dragline samples from females with different sexual excitation index (SEI). The *y*-axis represents the signal of individual peaks. Labeling of peaks: method/retention time.

Analysis of variance (ANOVA) showed significant differences in eleven peaks between groups of females with different sexual index (Figure 9). In order to reveal the ordinal character of the variable, the influence of the sexual excitation index was also investigated using ordinal logistic regression. This analysis identified two peaks (A250 2.99 min; A250 3.25 min) as significant, which indicates that not only is there a difference between groups, but the concentration of these substances also increases with increasing value of the index.

## 4. Discussion

This study investigated the influence of female draglines on male sexual behavior in the nursery-web spider, *P. mirabilis*. Male behavior was examined with behavioral tests, while chemical differences in draglines were examined with HPLC. Our results showed the complementarity of these approaches in some cases, while certain other treatments do not match when both behavioral and chemical data are considered.

Our first hypothesis (H1) dealt with males’ ability to discriminate between subadult and adult females. Both behavioral and HPLC data (H3) seem to be matching in this case, given that discrimination between the sexually receptive (adult) and nonreceptive (subadult) female is crucial for successful reproduction; males, indeed, produced nuptial gifts in the presence of draglines of subadult females less frequently than in the presence of adult females. Our findings align precisely with the experimental evidence presented by Eberhard et al. [66], which demonstrated that male *P. mirabilis* exhibited reduced inclination to engage in vibrational courtship and generated fewer pulses when interacting with the silk produced by subadult females as opposed to that produced by adult females. Discrimination of sexually receptive females is especially important in species with nuptial feeding such as *P. mirabilis*, where gift production is energetically costly and gift carrying impairs male locomotion [63,67]. Our results perfectly fit with experiments showing that males of *P. mirabilis* were less likely to perform vibrational courtship and produced fewer pulses when contacting the silk of subadult females compared to the silk produced by adult females [66].

Hungry *P. mirabilis* females are more sexually receptive than well-fed females, at least under laboratory conditions [48,55]. The high willingness to mate is explained by receiving direct benefits in terms of a dead prey wrapped with silk (nuptial gift). This strategy may be adaptive for females because it meets their foraging needs [58]. We suggested that females can actively signal their hunger level by pheromones in draglines to secure more matings. Superfluous matings can be costly, however, for females in terms of reduced reproductive success [68]. If hungry females avoid additional copulations, then male ability to discriminate between hungry and well-fed females is driven by male sexual exploitation rather than by female mate choice. Both of these possibilities predict differences in the chemical composition of draglines between hungry and fed females, although HPLC analyses failed to show significant differences (H4). Surprisingly, however, behavioral tests showed that males were able to correctly discriminate between fed and hungry females, because the draglines of the former group of females stimulated nuptial gift production significantly less than the draglines of the latter group of females. In contrast to the discrimination between subadult and adult females discussed above, these results show a small agreement between the behavioral and chemical data.

We unexpectedly found, however, significant differences in HPLC analyses of draglines between virgin and mated females. This difference is surprising not only because there was no agreement with the behavioral tests, as males did not produce nuptial gifts in the presence of virgin females more than in the presence of mated females (or vice versa), but also because previous work failed to find any behavioral differences (e.g., length of precopulatory phase, copulation duration) between males mated with virgin or with mated females (e.g., [66,68,69,70]). One plausible explanation is that the males were simply unable to respond to differences between the draglines of virgin and mated discovered by HPLC. Another alternative is that males are sensitive to these cues but ignore them, because they can still monopolize paternity via long mating [71]. 

Eggsac-carrying females produced sexually attractive pheromones that do not support our hypothesis (H5), although the chemical differences between the draglines of eggsac-carrying and not-carrying adult females were significant (H6). Males have a short lifespan relative to females (e.g., [56,58,62]) and there is a low chance of interactions between males and eggsac-carrying females in nature. Some male spiders can destroy female eggsacs to entice them to copulate [72], but this is not the case with *P. mirabilis* (P. Prokop, pers obs.). We suggest that selection does not need to operate against the production of sexually attractive pheromones even when female sexual receptivity declines in the case where these pheromones are not costly to produce.

Investment by males in nuptial gift production was not influenced by the level of hunger or the mating status of females. The presence of a rival male (high risk of sperm competition) in the mating arena was found to decrease the investment of males in gift production and the amount of sperm [73]. One could argue that the males in our study should reduce gift construction in the presence of the pheromones of mated females, because of the high risk of sperm competition. We suggest that the absence of visual cues of the risk of sperm competition is responsible for the null effect. Regarding the hunger level, Solano-Brenes et al. [74] also did not record differences in the silk investment in nuptial gifts by males interacting with fed and hungry females in the gift-giving spider *Paratrechalea ornata* (Mello-Leitão, 1943). Overall, male response rates on draglines were low, indicating that visual cues also play a crucial role in reproduction of this species [74].

## 5. Conclusions

This study showed that the mere presence of female draglines has a signaling function because it alters male mating behavior. The chemical signals of adult (versus subadult) and hungry (versus fed) females stimulated male motivation to mate, and highly sexually excited males invested more silk in gift production than less sexually excited males. HPLC helped us identify chemical differences in female draglines, but these differences did not always correspond to male behavior. Future research should attempt to identify additional types of HPLC in order to secure greater integration of the behavioral and chemical approaches to studying spider behavior.

## Figures and Tables

**Table 1:** Multiple logistic regression on the likelihood of gift production by males. CI are confidence intervals.

	Estimate	Wald’s χ^2^	−95% CI	+95% CI	*p*
Intercept	1.37	1.94			
Male mobility	0.10	1.13	0.92	1.33	0.29
Female mobility	0.09	2.20	0.97	1.23	0.14
Sexual excitement index	−1.00	6.93	0.17	0.77	0.008
Hunger level (fed)	0.87	3.93	1.02	32.37	0.047
Mating status (virgin)	−0.003	0.00005	0.15	6.48	0.99

**Table 2:** Generalized linear model with Tweedie distribution on male investment in gift production.

	Estimate	Wald’s χ^2^	−95% CI	+95% CI	*p*
Male mobility	−0.09	0.93	−0.27	0.09	0.34
Female mobility	−0.09	2.72	−0.21	0.018	0.10
Sexual excitement index	0.86	5.83	0.16	1.56	0.02
Hunger level (fed)	−0.55	1.64	−1.40	0.29	0.12
Mating status (virgin)	−0.15	0.09	−1.14	0.84	0.77

## Data Availability

Data will be available on the web page of the corresponding author at: http://thrips.katalogdruhov.sk/publications/prokop on 1 October 2023.
